# Hemodynamic evaluation in patients with transposition of the great arteries after the arterial switch operation: 4D flow and 2D phase contrast cardiovascular magnetic resonance compared with Doppler echocardiography

**DOI:** 10.1186/s12968-016-0276-8

**Published:** 2016-09-22

**Authors:** Kelly Jarvis, Marleen Vonder, Alex J. Barker, Susanne Schnell, Michael Rose, James Carr, Joshua D. Robinson, Michael Markl, Cynthia K. Rigsby

**Affiliations:** 1 Department of Radiology, Feinberg School of Medicine, Northwestern University, 737 North Michigan Avenue Suite 1600, Chicago, IL 60611 USA; 2 Department of Biomedical Engineering, McCormick School of Engineering, Northwestern University, Chicago, USA; 3 Center for Medical Imaging-North East Netherlands, University of Groningen, University Medical Center Groningen, Groningen, The Netherlands; 4 Department of Medical Imaging, Ann & Robert H. Lurie Children’s Hospital of Chicago, Chicago, USA; 5 Department of Pediatrics, Feinberg School of Medicine, Northwestern University, Chicago, USA; 6 Division of Cardiology, Ann & Robert H. Lurie Children’s Hospital of Chicago, Chicago, USA

**Keywords:** 4D flow CMR, 2D phase contrast CMR, Peak velocity, Transposition of the great arteries, Stenosis

## Abstract

**Background:**

Peak velocity measurements are used to evaluate the significance of stenosis in patients with transposition of the great arteries after the arterial switch operation (TGA after ASO). 4D flow cardiovascular magnetic resonance (CMR) provides 3-directional velocity encoding and full volumetric coverage of the great arteries and may thus improve the hemodynamic evaluation in these patients. The aim of this study was to compare peak velocities measured by 4D flow CMR with 2D phase contrast (PC) CMR and the gold standard Doppler echocardiography (echo) in patients with TGA after ASO.

**Methods:**

Nineteen patients (mean age 13 ± 9 years, range 1–25 years) with TGA after ASO who underwent 2D PC CMR and 4D flow CMR were included in this study. Peak velocities were measured with 4D flow CMR in the aorta and pulmonary arteries and compared to peak velocities measured with 2D PC CMR and Doppler echo. 2D PC CMR data were available in the ascending aorta, main, right and left pulmonary arteries (AAO/MPA/RPA/LPA) for 19/18/17/17 scans, respectively, and Doppler echo data were available for 13/9/6/6 scans, respectively. Peak velocities were measured with: 1) a single cross section for 2D PC CMR, 2) velocity maximum intensity projections (MIPs) for 4D flow CMR and 3) Doppler echo.

**Results:**

Significantly higher peak velocities were found with 4D flow CMR than 2D PC CMR in the AAO (*p =* 0.003), MPA (*p =* 0.002) and RPA (*p =* 0.005) but not in the LPA (*p =* 0.200). No difference in peak velocity was found between 4D flow CMR and Doppler echo (*p >* 0.46) or 2D PC CMR and echo (*p >* 0.11) for all analyzed vessel segments.

**Conclusions:**

4D flow CMR evaluation of patients with TGA after ASO detected higher peak velocities than 2D PC CMR, indicating the potential of 4D flow CMR to provide improved stenosis assessment in these patients.

## Background

Pulmonary artery stenosis either at the anastomosis or in the branch pulmonary arteries (PA) is the most common complication leading to intervention after the arterial switch operation (ASO) for transposition of the great arteries (TGA) [1–3]. Accurately depicting PA stenosis is therefore paramount for a postoperative TGA evaluation. However, standard tools such as 2D phase contrast cardiovascular magnetic resonance (2D PC CMR) or Doppler echocardiography (echo) rely on velocity quantification in a single imaging plane with uni-directional velocity encoding and may not accurately detect the peak velocity across entire vessel segments. Furthermore, the complex vascular geometry following ASO and limited acoustic windows complicates interrogation with Doppler echocardiography, especially in older children.

Three-dimensional (3D) cine (time-resolved) phase contrast CMR with 3-directional velocity encoding (4D flow CMR) [4] provides full volumetric coverage of the great arteries and may thus improve hemodynamic evaluation in complex post-surgical anatomy. The 4D flow CMR technique is useful for the assessment of 3D blood flow characteristics and the retrospective analysis of regions of interest in the heart and surrounding vessels [5–8]. Previous studies [9–13] have demonstrated excellent flow parameter agreement and improved volumetric velocity analysis when using 4D flow CMR compared to 2D PC CMR. In addition, several studies [14–16] have been performed to assess the reliability of flow measurements. However, to the best of our knowledge, no study has focused on the evaluation of peak velocity measured by 4D flow CMR in patients with TGA. Our aim was to compare peak velocities measured by 4D flow CMR with 2D PC CMR and the non-invasive gold standard Doppler echo in patients with TGA after ASO.

## Methods

### Population

Twenty-three patients with D-TGA after ASO were included in this Institutional Review Board (IRB) approved and HIPAA compliant study. The study was approved by the IRB of Ann & Robert H. Lurie Children’s Hospital and Northwestern University. All patients underwent a standard clinically indicated CMR exam including 2D PC CMR. Twenty-two subjects were prospectively recruited to undergo additional 4D flow CMR and written informed consent was obtained from all participants. One patient who underwent cardiac CMR including 4D flow CMR as standard-of-care was included via retrospective chart review as approved by the IRB.

### Image acquisition and analysis

2D PC CMR and 4D flow CMR acquisitions were performed on either a 1.5 T Avanto or Aera MR scanner (Siemens, Erlangen, Germany) during the same exam as the clinically indicated cardiac CMR study. General anesthesia was utilized per the clinical CMR protocol.

#### 4D Flow CMR

Whole heart 4D flow CMR was performed (spatial resolution = 1.8–4.1 × 1.3–2.7 × 1.5–3.0 mm^3^, FOV = 250-340 × 125–298 mm^2^, slab thickness = 72–134 mm, temporal resolution = 36.8–43.2 ms, TE = 2.3–2.8 ms, TR = 4.6–5.4 ms, flip angle = 15°, bandwidth = 450 Hz/pixel, velocity sensitivity (venc) = 100–200 cm/s) with full volumetric coverage of the great arteries. The average scan time was 11 min.

**Fig. 1:**
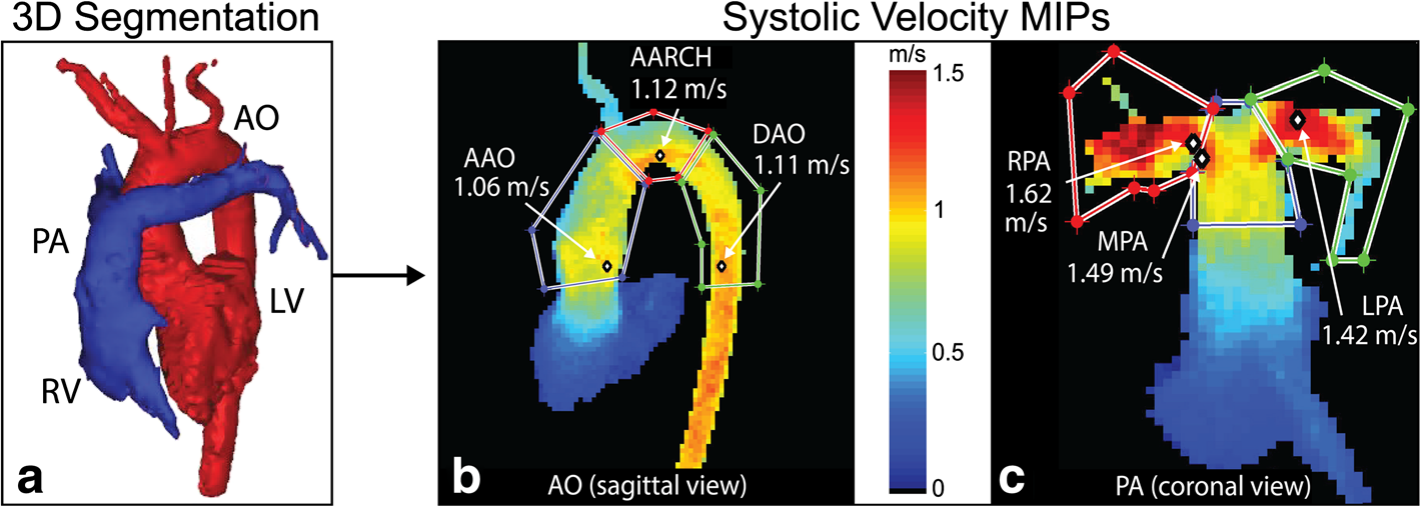
Methods workflow. **a** The aorta (*red*) and pulmonary (*blue*) volumes were segmented and separated from each other. The MIPs were viewed in 3 orientations (only one is shown here) and regions of interest were drawn to determine maximum velocities (*white* circles with *black* border) in the **b** aorta (AAO, AARCH, DAO) and **c** MPA, RPA and LPA

In-house developed software (Northwestern University Radiology, Chicago, USA) was used for the processing of 4D flow CMR data for correcting for Maxwell terms, eddy currents, and velocity aliasing as previously described [17, 18]. The time averaged 3D phase contrast angiogram (PC-MRA) was calculated from the 4D flow CMR data to depict the anatomy of the heart and surrounding vessels. The aorta and pulmonary arteries were manually segmented in 3D from the rest of the imaging data using commercial software (Mimics Innovation Suite, Materialise, Belgium) (Fig. 1a). The peak velocities were determined using maximum intensity projections (MIPs) of the aorta and pulmonary arteries (Matlab, MathWorks, USA) [19]. MIPs were used to show peak velocity (using the magnitude of the three-directional velocity vectors) in the volume and over multiple time-points projected onto a 2D viewing plane. Only velocity data from time-points covering peak systole were included in the MIPs. By using three different viewing planes (sagittal, coronal, axial), 3D regions of interest (ROIs) were identified. The peak velocity magnitude was determined within each 3D ROI. 4D flow CMR results in the ​ascending aorta (AAO), main pulmonary artery (MPA), right pulmonary artery (RPA), and left pulmonary artery (LPA) (Fig. 1b-c) were compared to 2D PC CMR and echo. Since 4D flow CMR allows for the retrospective analysis of the entire vessel, regions of interest were also generated for the aortic arch (AARCH) and descending aorta (DAO) (Fig. 1b).

To remove noisy voxels near the boundary of the vessel lumens, segments were eroded by one voxel prior to analysis, unless the size of the vessel was so small that this was not feasible. If needed, regions of interest could be adjusted to remove noisy voxels by the boundary. To account for any remaining noisy voxels a noise filter was applied that looked in the ROI for large shifts in peak velocity value [19]. Voxels associated with large incremental shifts were considered noise and removed.

Peak velocity MIP analysis was performed by two observers, blinded to each other’s results. This inter-observer study included the drawing of 3D regions of interest (AAO, AARCH, DAO, MPA, RPA and LPA) as well as determining the level of volumetric erosion applied.

#### 2D PC CMR

In order to obtain the most accurate peak velocity measurement position in the LPA and RPA, a 2D PC CMR acquisition was first acquired in the plane of the vessel of interest (in-plane) at a velocity encoding setting intended to allow aliasing in the location of peak flow velocity. The aliasing location was used to guide placement of a slice acquired perpendicular (through-plane) to the vessel of interest in the region of the highest velocity. For LPA and RPA peak velocity measurements in patients without in-plane positioning, the through-plane 2D PC CMR slice was acquired in a location where the highest velocity was suspected. The imaging was prescribed to have a line of at least four pixels (i.e. approximately 16 voxels covering the vessel lumen) along the diameter of each vessel [20]. For the AAO and MPA measurements, imaging slices were placed at the levels of the sinotubular junctions during systole. Imaging parameters for the through-plane 2D PC CMR: spatial resolution = 0.9–2.0 mm^2^, FOV = 135–344 × 180–379 mm^2^, slice thickness = 5–6 mm, temporal resolution = 10.8–49.0 ms, TE = 1.9–4.4 ms, flip angle = 15–30°, bandwidth = 360–600 Hz/pixel, venc = 120–420 cm/s, number of averages = 1–3; and for in-plane imaging: spatial resolution = 1.1–1.6 mm^2^, FOV = 165–290 × 220–320 mm^2^, slice thickness = 5 mm, temporal resolution = 20.6–28.0 ms, TE = 3.1–4.4 ms, flip angle = 20°, bandwidth = 480–600 Hz/pixel, venc = 200–420 cm/s, number of averages = 1–3. Scan times were typically 1–3 min per scan with four 2D PC CMR scans (aorta, pulmonary artery, right and left branch pulmonary arteries) generally run for each patient. Post processing for peak velocities was performed by placing a region of interest surrounding the vessel of interest using QFlow software (Medis, Leiden, The Netherlands).

#### Doppler echocardiography

For patients who underwent routine, clinically indicated echo within 1 year of the CMR exam, maximum instantaneous velocities were measured retrospectively from best available spectral Doppler imaging. All studies were performed on Philips IE33 ultrasound machines (Philips Healthcare, Best, Netherlands) using the optimal transducer for patient size. Peak velocity was obtained by continuous wave Doppler recordings from the apical or suprasternal window for the aorta and from the parasternal or suprasternal windows for the main and branch PAs.

### Statistical analysis

Wilcoxon matched-paired signed-rank test was used to calculate the difference of peak velocity between 2D PC CMR, 4D flow CMR and Doppler echo measurements (α = 0.05). Agreement between measurement techniques and between observers was evaluated by Bland-Altman analysis.

## Results

### Patient population

**Fig. 2:**
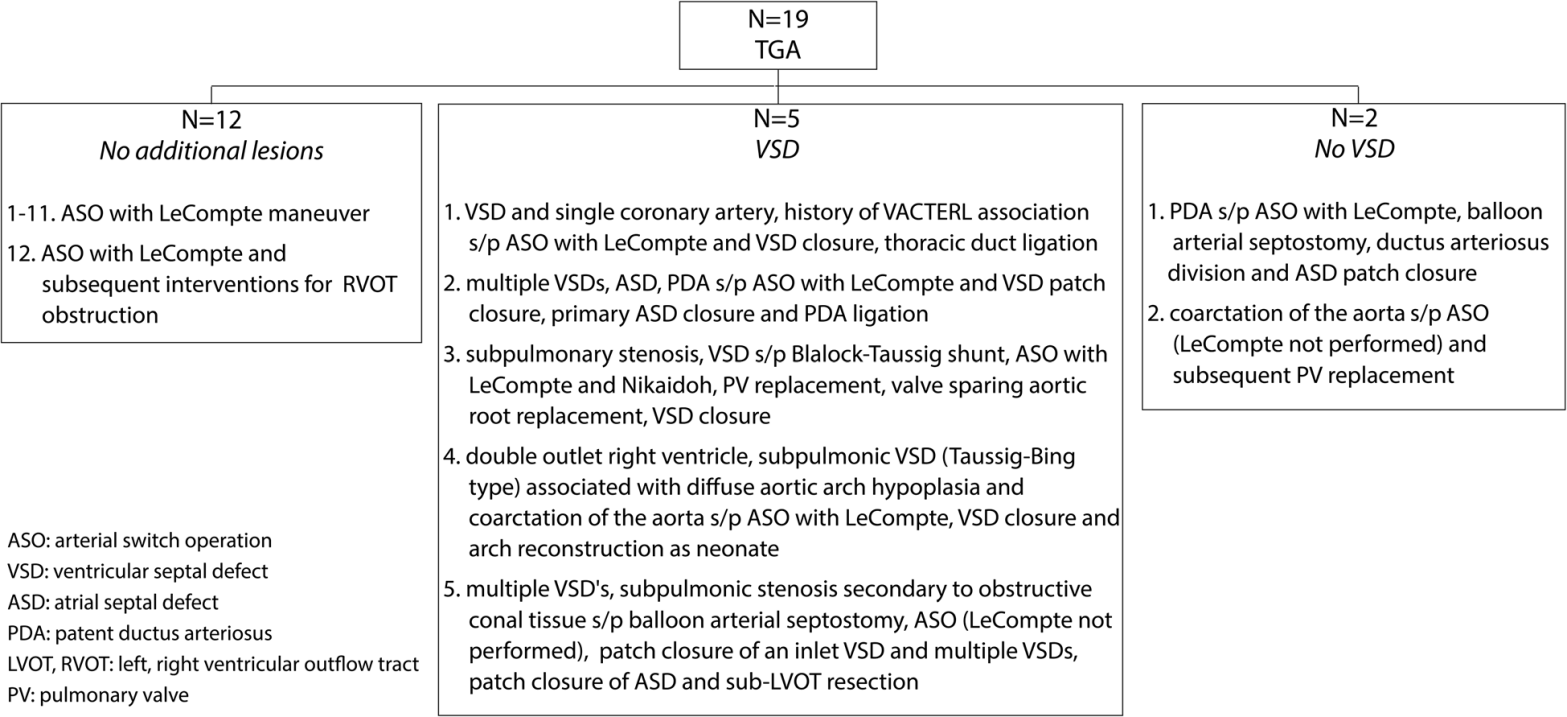
Patient cohort diagnosis and surgical history

Twenty-three patients with TGA after ASO underwent 4D flow CMR in addition to their standard clinically indicated CMR. Four patients had 4D flow CMR acquisitions that contained aliased velocity regions, which could not be corrected by automatic or manual techniques; therefore, they were excluded from the study. Nineteen patients were included in the final cohort (Fig. 2) with a mean ± SD age of 13 ± 9 years (range 1–25 years) and 68 % male. Eleven patients underwent anesthesia for the CMR scan as per the institutional clinical protocol. 2D PC CMR was performed in these patients as part of the clinical examination. The patients were assessed as part of their ongoing follow-up care and for clinical indications including pulmonary artery stenosis, ventricular size and function, aortic dimensions, aortic and pulmonary regurgitant flow, differential pulmonary blood flow, coronary arteries and neoaortic valve function. Two patients did not have 2D PC CMR data available for the PAs in all regions of interest. Echo data within 1 year of the CMR scan was available in the AAO/MPA/RPA/LPA for 13/9/6/6 patients (68 %/47 %/32 %/32 %), respectively. The mean time between the echo and CMR exams was 4.2 ± 2.9 months (range 0.2–10.6 months).

### Peak velocity comparison

**Fig. 3:**
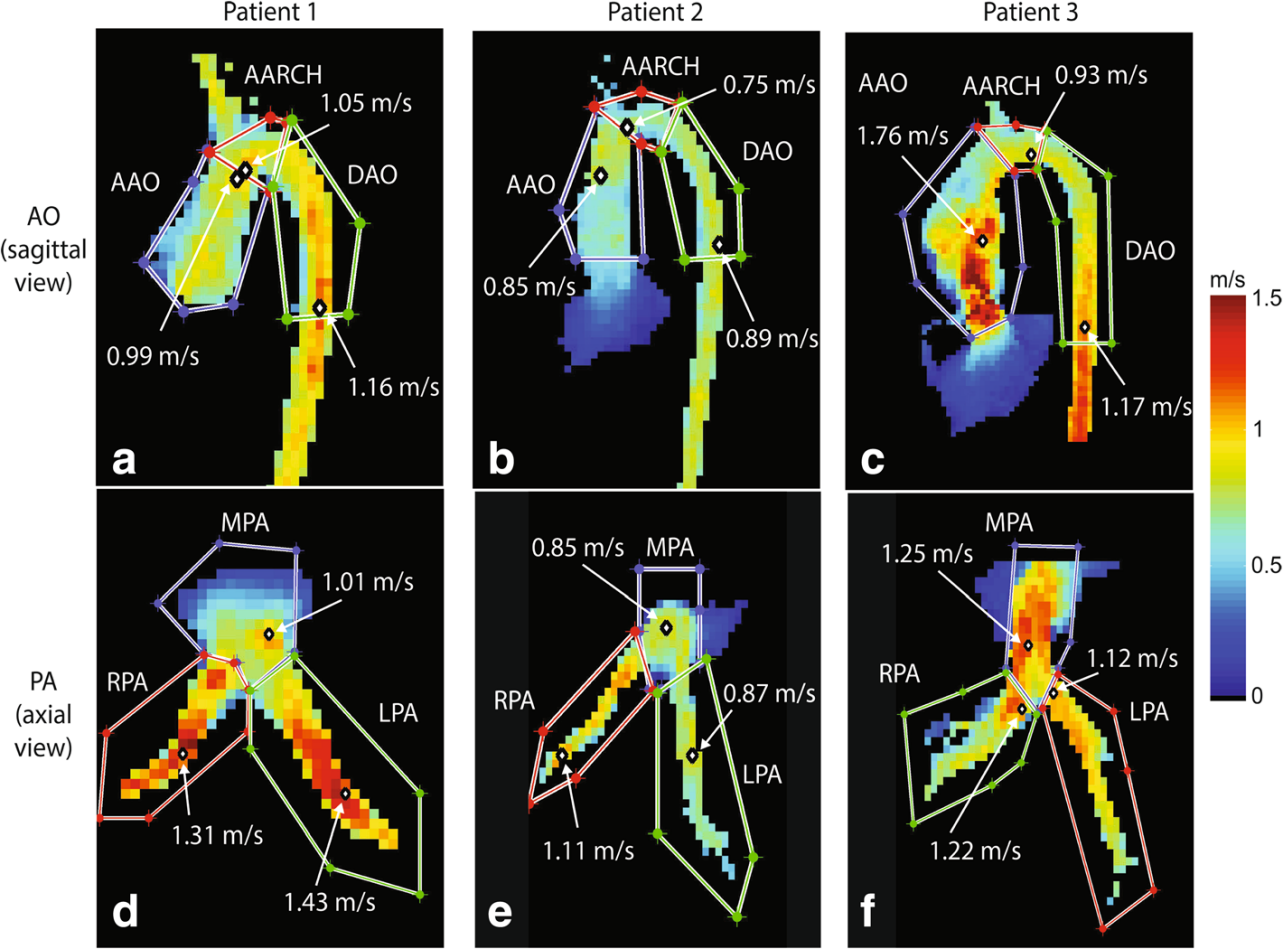
Examples of maximum velocity results. MIP images of the aorta (**a**-**c**) and the pulmonary artery (**d**-**f**) in three patients (left, middle, right) with TGA after ASO and the corresponding maximum velocity in regions of interest

**Fig. 4:**
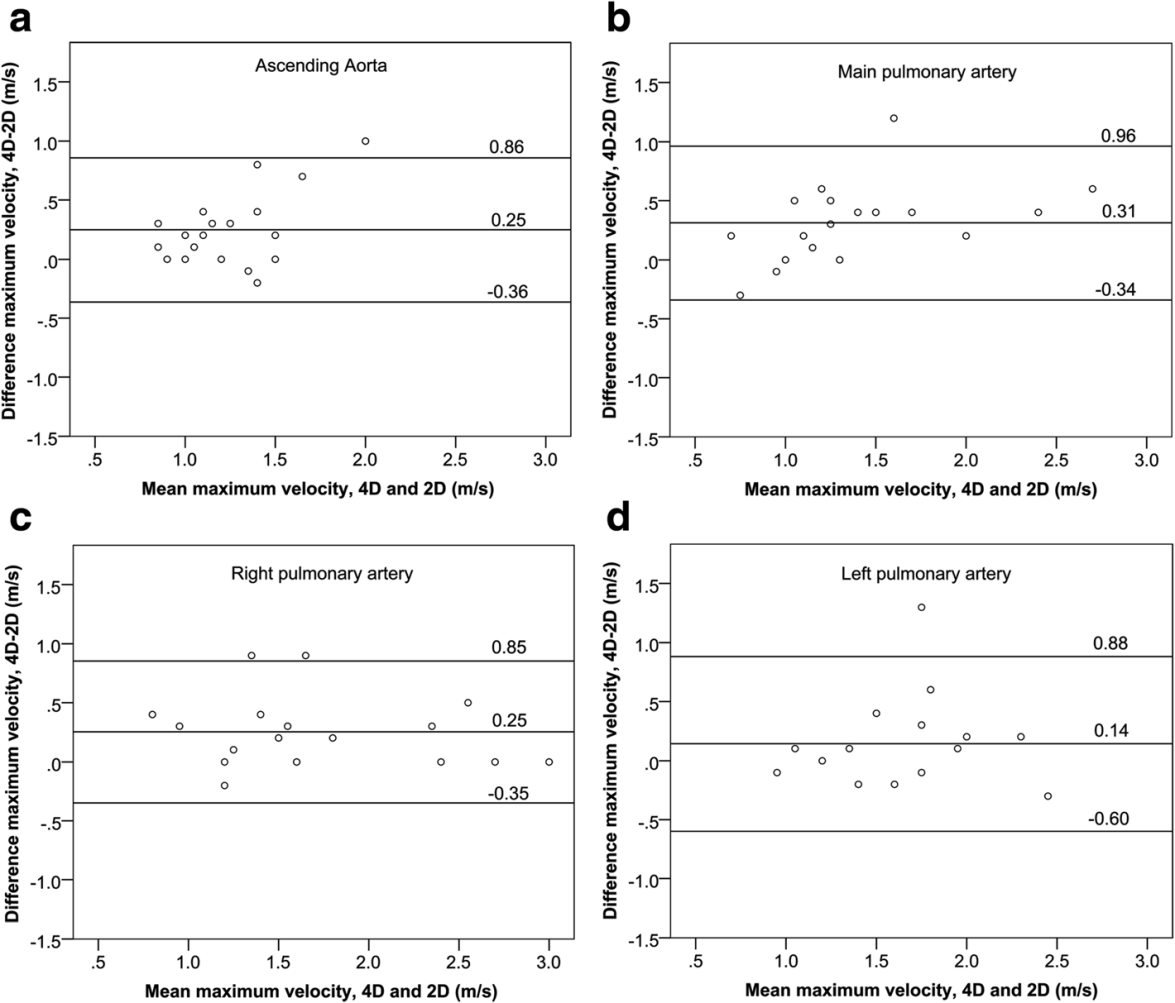
Bland-Altman plots for maximum velocities obtained in the **a** AAO, **b** MPA, **c** RPA and **d** LPA

**Fig. 5:**
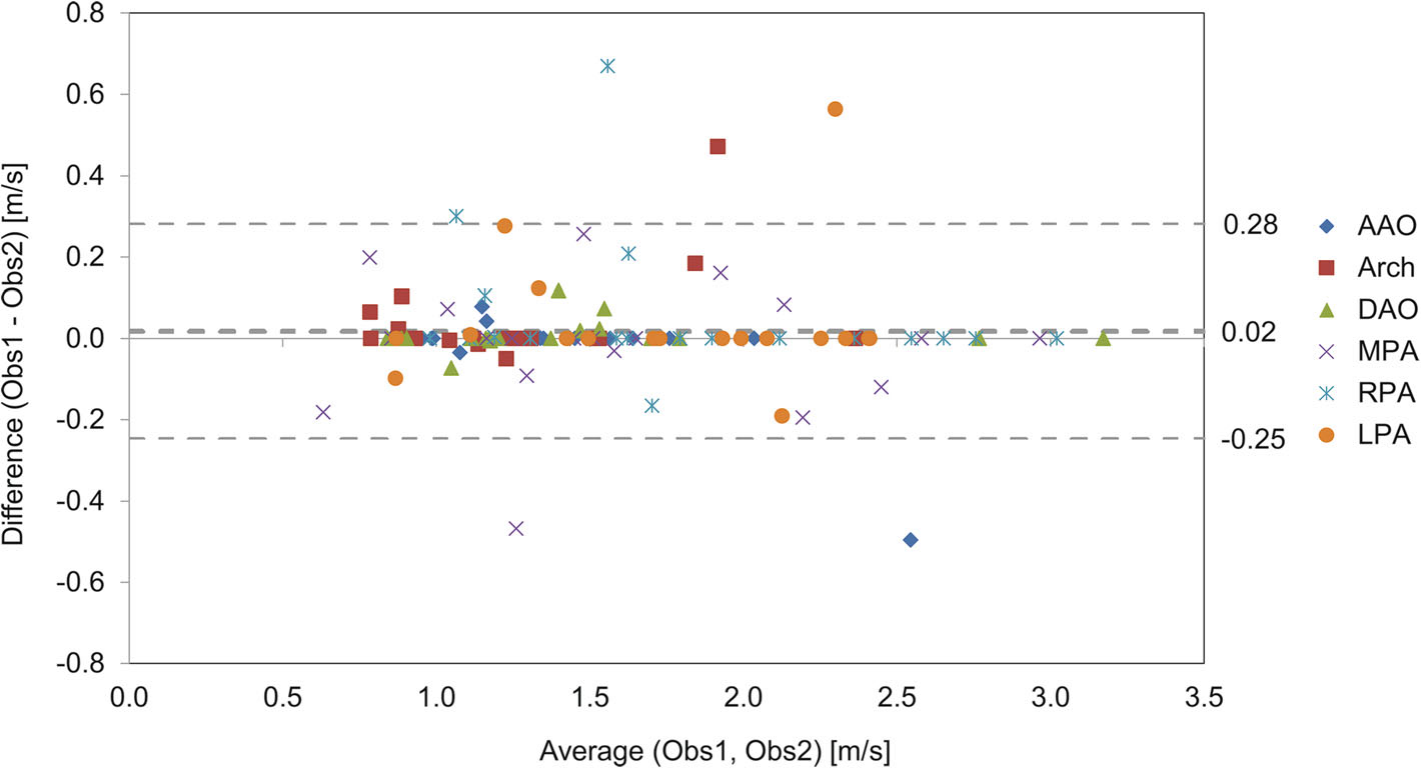
Bland-Altman plot for the 4D flow CMR inter-observer study

Comparison of peak velocities (Table 1) shows significantly higher peak velocities with 4D flow CMR (Fig. 3) than with 2D PC CMR for the AAO, MPA and RPA but not for the LPA. Bland-Altman results (Fig. 4) showed 4D flow CMR peak velocity measurements were higher than those by 2D PC CMR with mean difference ranging from 0.14 m/s to 0.31 m/s in the analyzed segments. No difference in peak velocity was found between 4D flow CMR and echo for all analyzed vessel segments (Table 2), nor was there a significant difference in peak velocity between 2D PC CMR and echo. The inter-observer study for 4D flow CMR peak velocity analysis showed good agreement with no sizable bias (0.02 m/s or 1 % of average value of 1.5 m/s) between observers and limits of agreement of −0.25 to 0.28 m/s (−16 to 18 % of the average value of 1.5 m/s) (Fig. 5).

**Table 1:** Comparison of peak velocities between 4D flow CMR and 2D PC CMR

	Sample size (n)	4D flow CMR peak velocity (m/s)	2D PC CMR peak velocity (m/s)	*p*-value
		mean (SD)	mean (SD)	
AAO	19	1.4 (0.4)	1.1 (0.3)	0.003
MPA	18	1.5 (0.6)	1.2 (0.5)	0.002
RPA	17	1.8 (0.6)	1.5 (0.7)	0.005
LPA	17	1.7 (0.5)	1.5 (0.5)	0.200

**Table 2:** Comparison of peak velocities of 4D flow CMR and 2D PC CMR and Doppler echo

	Sample size (n)	4D flow CMR peak velocity (m/s) mean (SD)	2D PC CMR peak velocity (m/s) mean (SD)	Doppler Echo peak velocity (m/s) mean (SD)	4D flow CMR & Doppler Echo *p*-value	2D PC CMR & Doppler Echo *p*-value
AAO	13	1.4 (0.5)	1.1 (0.3)	1.5 (0.7)	0.824	0.114
MPA	9	1.5 (0.7)	1.2 (0.5)	1.5 (0.5)	0.858	0.139
RPA	6	2.0 (0.8)	1.8 (0.8)	2.0 (0.6)	0.833	0.600
LPA	6	1.8 (0.5)	1.6 (0.5)	2.1 (0.8)	0.463	0.207

**Fig. 6:**
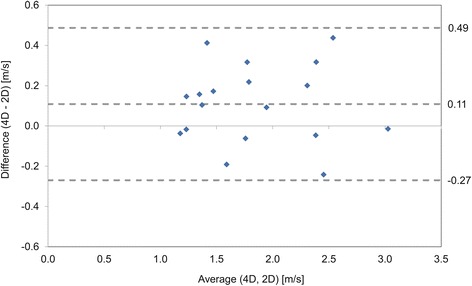
Bland-Altman plot for only scans where the 2D PC CMR through-plane location was guided by in-plane imaging

The location of through-plane 2D PC CMR was guided by in-plane imaging in 9 patients for the LPA and RPA. Considering only these scans and combining the through-plane imaging results for the RPA and LPA regions, there was a statistically significant difference between 4D flow CMR and 2D PC CMR (*p =* 0.050, *n =* 18, mean ± SD = 4D flow CMR: 1.89 ± 0.56 vs. 2D PC CMR: 1.79 ± 0.55 m/s). Three of the 9 patients had corresponding echo data. No difference was detected between 4D flow CMR and echo or 2D PC CMR and echo (*p =* 0.29 and *p =* 0.60 respectively, *n =* 6). Bland-Altman analysis (Fig. 6) showed higher peak velocities from 4D flow CMR than 2D PC CMR with a bias of 0.11 m/s and limits of agreement between −0.27 and 0.49 m/s. In percentages, this is a bias of 6 % and limits of agreement between −15 and 26 % of the average measurement (1.8 m/s). When looking at the LPA and RPA data separately, a statistically significant difference was still detected between the 4D flow CMR and 2D PC CMR measurements in the RPA (*p =* 0.027) but not for the LPA (*p =* 0.72).

## Discussion

We found significantly higher peak velocities with 4D flow CMR than 2D PC CMR for 3 out of the 4 analyzed regions of interest, indicating the potential of 4D flow CMR to provide improved stenosis assessment in the pulmonary arteries in patients with TGA after ASO. No difference was found in peak velocities between 4D flow CMR and echo, or 2D PC CMR and echo, for any of the analyzed vessels. The small number of patients with echo data could be the reason why no significant difference in peak velocity was found when comparing to echo, particularly in the LPA and RPA (*n =* 6).

There are two main explanations for higher peak velocity detection using 4D flow CMR versus 2D PC CMR: 1) 2D PC CMR is typically evaluated at the aortic and pulmonary roots or in areas where stenosis is suspected (e.g. LPA and RPA) and is therefore limited to velocities in the 2D cross section of the selected region. The 2D PC CMR plane placement was based on the anatomy of the vessel, unless in-plane imaging was used, which was the case for about half the LPA and RPA measurements. 4D flow CMR enables the retrospective analysis of regions of interest in the imaging volume (containing the heart and large vessels) and detects peak velocities in the entire vessel. 2) With 2D PC CMR, the velocity is measured in one direction normal to the imaging plane. 4D flow CMR measures velocity in three directions (v_x_, v_y_ and v_z_) to determine the magnitude of the velocity vector in any direction and to account for eccentric flow jets that cannot be accounted for with 2D PC CMR. We have previously found through-plane velocity to significantly underestimate velocity magnitude compared to three-directional velocity encoding [13, 19].

Reintervention rates are low following ASO, however, velocity quantification is an important factor in determining indications for reintervention in the case of pulmonary artery stenosis, dilatation of the aortic root and valve insufficiency [1, 6]. 4D flow CMR peak velocity MIP analysis allows for the improved visualization of peak velocities for vessels of interest in patients with TGA after ASO. This was also seen in a study of Robinson et al. (2014), in which MIPs allowed efficient and improved visualization of residual right ventricular outflow tract obstruction in patients with tetralogy of Fallot [21]. Moreover, with 4D flow CMR analysis of multiple flow characteristics is possible in TGA patients. A study by Geiger et al. (2014) showed that with 4D flow CMR, anomalous flow patterns can be revealed in TGA patients [6]. The analysis of flow patterns together with the quantitative information of the peak velocity and novel hemodynamic parameters like shear stress, vortex formation and pressure gradients, could provide a powerful tool in the post-surgical long-term follow-up of TGA patients.

To our knowledge, this is the first study in patients with TGA after ASO which systematically compares the peak velocity by 4D flow with conventional non-invasive methods. In a previous study by our group Gabbour et al. (2015), peak velocities based on 4D flow CMR and 2D PC CMR were compared in patients with various (corrected) cardiac congenital malformations. Similar to our current findings, peak velocity was underestimated by 2D PC CMR compared to 4D flow CMR for the aorta and main pulmonary artery [13]. Although these results indicate peak velocity is underestimated by 2D PC CMR, a study of Nordmeyer et al. (2010) showed that there are no significant differences in stroke volume and flux curves between 4D flow CMR and 2D PC CMR in healthy volunteers, and no significant differences for measuring antegrade and retrograde flow in congenital heart disease patients [9]. Therefore, in clinical practice, one should be aware of the specific parameter employed for a particular PC CMR technique, to allow adequate decision-making based on quantitative information.

The acquisition of CMR and echo occurred at separate times, and anesthesia was used per the clinical protocol for some CMR exams but not for any Doppler echo measurements. We have limited the time between CMR and echo to 1 year to mitigate the effects from stenosis progression over time. However, that the exams were not performed on the same day and that some patients underwent anesthesia for one technique and not the other remains a limitation.

The temporal resolution for the through-plane 2D PC CMR was on average 23.9 ms, but lower temporal resolutions (34.8–49.0 ms) were utilized in some patients to acquire data within one breath hold. These lower temporal resolution values were comparable to those of 4D flow CMR (36.8–43.2 ms), but this still is a study limitation.

Even with the imaging acceleration of GRAPPA (R = 2) and k-t GRAPPA (R = 5), a wide range of spatial resolutions were needed to accommodate varying patient size and keep 4D flow CMR scan times on the order of 5–10 min. Low spatial resolution may lead to an underestimation of peak velocity from partial volume effects when high velocities regions are averaged with low velocity regions within the same voxel. Nevertheless and despite the lower resolution, 4D flow CMR velocities were comparable to echo and higher than 2D PC CMR.

Because only a single imaging plane is set at the time of image acquisition in a vessel segment with 2D PC CMR, the resulting measurements are operator-dependent. With 4D flow CMR a whole volume is scanned, minimizing the role of the operator during acquisition. However, the 4D flow CMR technique requires multiple post-processing steps before the peak velocity can be determined in a MIP. While MIP post-processing showed good agreement between observers, post-processing of the entire case by different operators might lead to a slightly different peak velocity that can be determined only from the 4D flow CMR velocity MIP. When differences were detected between observers during the MIP post-processing they were generally due to 1) differences in the way the regions of interest were drawn (since this is in 3D there is an added level of difficulty regions must be drawn in all three views) and 2) differences in the level of volumetric erosion used on the vessel segment.

It would be interesting to test whether the detection rate of stenosis improves with 4D flow CMR, given the ability of 4D flow CMR to detect higher velocities with volumetric coverage and 3-directional velocity encoding. Pulmonary branch stenosis is typically the concern for these patients. However, there is no clear consensus regarding a maximum velocity cutoff for definitively determining stenosis in the pulmonary artery branches with 2D PC CMR in these patients. In fact, when grading or considering reintervention for branch PA stenosis following the LeCompte manueuver, there are a number of other factors to consider, including patient pulmonary artery anatomy, differential pulmonary blood flow, the difficulty of stenting complex PA geometry and the attendant risk of complications following potential intervention. Invasive catheter pressure measurements would be helpful for comparison. However, our patients were not considered to have stenosis severe enough to warrant intervention following their MR examinations. Now that we have determined that we can detect higher velocities with 4D flow than 2D PC CMR, future studies are warranted in larger cohorts to determine the clinical impact of 4D flow on stenosis detection.

## Conclusions

Using 4D flow CMR velocity MIPs in patients with TGA after ASO enables the quantification of peak velocity in the aorta and pulmonary arteries and may provide an improved depiction of vessel stenosis. Our 4D flow CMR results were comparable to echo and detected higher peak velocities than 2D PC CMR, even when compared to cases where in-plane 2D PC CMR was used to refine velocity quantification in an area of stenosis. Larger studies are needed to confirm these findings and to determine the clinical impact of stenosis quantified by 4D flow CMR peak velocity MIPs.

## Abbreviations

3D: Three-dimensional
4D flow CMR: Three-dimensional cine phase contrast CMR with 3-directional velocity encoding
AAO: Ascending aorta
AARCH: Aortic arch
ASO: Arterial switch operation
CMR: Cardiovascular magnetic resonance
DAO: Descending aorta
Echo: Doppler echocardiography
LPA: Left pulmonary artery
MIP: Maximum intensity projections
MPA: Main pulmonary artery
PA: Pulmonary artery
PC-MRA: Phase contrast magnetic resonance angiogram
RPA: Right pulmonary artery
TGA: Transposition of the great arteries
Venc: Velocity sensitivity
